# Author’s Responses to Peer Reviews of “Forecasting the COVID-19 Pandemic in Saudi Arabia Using a Modified Singular Spectrum Analysis Approach: Model Development and Data Analysis”

**DOI:** 10.2196/28742

**Published:** 2021-03-31

**Authors:** Nader Alharbi

**Affiliations:** 1 King Saud bin Abdulaziz University for Health Sciences King Abdullah International Medical Research Center Riyadh Saudi Arabia

**Keywords:** COVID-19, prediction, singular spectrum analysis, separability, eigenvalues, Saudi Arabia

*These are the author's response to peer reviews for “Forecasting the COVID-19 Pandemic in Saudi Arabia Using a Modified Singular Spectrum Analysis Approach: Model Development and Data Analysis”.*

## Response to Round 1 Reviews

First, I wish to thank the editor and all the reviewers for their enlightening comments and observations, which I strongly believe have increased the quality of this manuscript [1]. I am indeed grateful for the time they have devoted to this paper. I hope you will be pleased to learn that I have taken on board and addressed all observations and comments.

### Anonymous [2]

#### Specific Comments

1. Thank you for this comment [2]. I have revised the discussion of the singular spectrum analysis technique and COVID-19 cases according to the referee’s suggestion, and I added more information and citations.
2. I appreciate the reviewer’s insightful comment. The test was used, and the results are provided. The recommended paper and another related paper were cited.

### Anonymous [3]

#### General Comments

Thank you for pointing out the formatting issues to me [3]. I agree, they must have been due to a formatting bug during submission. I hope now you will be pleased, as I have addressed all observations and submitted the paper as a Word file.

## Response to Round 2 Review

I appreciate the reviewer’s insightful comments and suggestions.

1. The whole manuscript has been reviewed and edited by a native speaker as suggested.

2. Moreover, the references have been removed from the Abstract and added to the Introduction section.
