# Correction: Author’s Responses to Peer Reviews of “Forecasting the COVID-19 Pandemic in Saudi Arabia Using a Modified Singular Spectrum Analysis Approach: Model Development and Data Analysis”

**DOI:** 10.2196/29879

**Published:** 2021-04-26

**Authors:** Nader Alharbi

**Affiliations:** 1 King Saud bin Abdulaziz University for Health Sciences King Abdullah International Medical Research Center Riyadh Saudi Arabia

In “Author’s Responses to Peer Reviews of ‘Forecasting the COVID-19 Pandemic in Saudi Arabia Using a Modified Singular Spectrum Analysis Approach: Model Development and Data Analysis’” (JMIRx Med 2021;2(1):e28742) one error was noted.

The reviewer IDs of two anonymous reviewers have been removed from the published article to preserve anonymity.

The correction will appear in the online version of the paper on the JMIR Publications website on April 26, 2021, together with the publication of this correction notice. Because this was made after submission to PubMed, PubMed Central, and other full-text repositories, the corrected article has also been resubmitted to those repositories.

